# Paquinimod Targeting of the S100A8/A9 Axis Suppresses Liver Metastasis in Aged Mice

**DOI:** 10.3390/cancers18101635

**Published:** 2026-05-19

**Authors:** Takao Tsuneki, Masafumi Saito, Kimihiro Yamashita, Masayuki Ando, Keisuke Yasuda, Naoto Shirakami, Ryota Ito, Yukari Adachi, Hiroki Kagiyama, Takaaki Tachibana, Masaki Imai, Sachiko Inubushi, Kazuki Kanayama, Yu-Ichiro Koma, Mitsugu Fujita, Joerg-Matthias Pollok, Yutaka Sugita, Taro Ikeda, Yasufumi Koterazawa, Tomoaki Aoki, Hitoshi Harada, Yasunori Otowa, Naoki Urakawa, Hironobu Goto, Hiroshi Hasegawa, Shingo Kanaji, Takeru Matsuda, Yoshihiro Kakeji

**Affiliations:** 1 Division of Gastrointestinal Surgery, Department of Surgery, Graduate School of Medicine, Kobe University, Kobe 650-0017, Hyogo, Japan; 2 Department of Immunology and Microbiology, National Defense Medical College, Tokorozawa 359-8513, Saitama, Japan; 3 Department of Biophysics, Graduate School of Health Sciences, Kobe University, Kobe 654-0142, Hyogo, Japan; 4 Division of Breast Surgery, Department of Surgery, Graduate School of Medicine, Kobe University, Kobe 650-0017, Hyogo, Japan; 5 Center for Medical Education and Clinical Training, Faculty of Medicine, Kindai University, Higashiosaka 577-8502, Osaka, Japan; 6 Division of Surgery and Interventional Science, Faculty of Medical Sciences, University College London, London NW3 2QG, UK

**Keywords:** aging, liver metastasis, hepatic immune microenvironment, myeloid immune cells, S100A8/A9 axis, paquinimod, CD8^+^ T cells

## Abstract

**Background:** Aging profoundly alters host immunity, yet how age-associated immune changes in the liver influence the growth of metastatic tumors remains incompletely understood. Liver metastasis is a major cause of cancer-related mortality, particularly in elderly patients, for whom aggressive treatments are often not feasible. This study aimed to clarify how aging reshapes the hepatic immune microenvironment and to identify age-associated host factors that influence liver metastasis growth. **Methods:** Tumor-naïve and tumor-bearing young and aged mice were analyzed using a syngeneic MC38 liver metastasis model. Immune cell composition in the liver was assessed by flow cytometry, and gene expression was evaluated by quantitative reverse transcription PCR (RT–qPCR). Public transcriptomic datasets were screened to identify age-associated inflammatory factors. The functional relevance of the S100A8/A9 axis was examined using the small-molecule inhibitor paquinimod. **Results:** Aging was associated with a distinct baseline immune cell composition in the liver. During liver metastasis, overall growth was comparable between young and aged mice; however, metastatic lesions in aged hosts showed increased expression of multiple inflammation-related genes and prominent accumulation of Ly6G^+^ cells. In silico screening identified *S100a9* as one of the most highly upregulated inflammation-related genes in aged livers, which was confirmed in both tumor-naïve and metastatic liver tissues. Pharmacological modulation of the S100A8/A9 axis with paquinimod significantly reduced liver metastasis growth in aged, but not young, mice, and was accompanied by a shift in immune cell composition, including an increased representation of CD8^+^ T cells. **Conclusions:** These findings indicate that aging is associated with a distinct hepatic immune context that shapes the inflammatory and cellular composition of the tumor microenvironment during liver metastasis. S100A9 emerges as a key age-associated, host-derived factor that is functionally relevant to the growth of liver metastases in aged hosts, supporting the S100A8/A9 axis as a context-specific therapeutic target.

## Simple Summary

As the population ages, an increasing number of cancer patients are elderly, yet treatment options for older patients are often limited because aggressive therapies may not be feasible. Liver metastasis is a major cause of cancer-related death, especially in older patients, but how the aging of the host influences the growth of metastatic tumors in the liver remains poorly understood. In this study, we show that aging is associated with characteristic changes in the immune environment of the liver, marked by increased accumulation of myeloid immune cells and inflammatory signals. We identify S100A9, an inflammation-related protein produced by myeloid cells, as a key factor that is elevated in aged livers and liver metastases. Importantly, pharmacological modulation of the S100A8/A9 axis reduced liver metastasis growth specifically in aged mice, but not in young mice. These findings suggest that age-related changes in the liver create a unique immune context that can be therapeutically targeted, providing a potential strategy for treating liver metastasis in elderly patients.

## 1. Introduction

Population aging has led to a steady increase in the number of older patients with cancer [1]. In elderly patients, curative-intent surgery and intensive multimodal therapies are often not feasible because of frailty, comorbidities, and limited physiological reserve [2,3]. As a result, treatment intensity is frequently attenuated and therapeutic options become restricted, particularly in the setting of advanced disease [4,5]. In this context, liver metastases represent one of the most clinically consequential events during the clinical course of many solid tumors and remain a major determinant of prognosis. Accordingly, achieving sustained disease control in the liver remains an important but challenging goal in the management of advanced cancer, especially in older patients for whom aggressive treatment strategies are often not applicable. These clinical realities highlight the importance of understanding how host-related factors influence the growth of liver metastases in the aging host.

One of the most prominent host-related changes associated with aging is a shift toward myeloid-biased hematopoiesis, leading to an increased supply of myeloid-lineage cells [6,7]. In cancer, such myeloid-skewed immune cell compositions are increasingly linked to the development of an immunosuppressive tumor microenvironment (TME) [8,9,10]. The liver is intrinsically a myeloid-rich and immunologically tolerogenic organ [11,12,13], suggesting that aging-associated myeloid bias may be particularly amplified in the hepatic immune environment. This raises the possibility that host aging substantially alters the immune cell composition of liver metastases and thereby influences their growth [14,15]. However, the molecular factors that drive the aging-associated, myeloid-skewed immune context in the liver remain poorly defined [16,17].

We focused on S100A9, a damage-associated molecular pattern protein that is highly expressed in activated myeloid cells [18,19]. S100A9 predominantly functions as a heterodimer with S100A8 (calprotectin), which represents the dominant biological form in myeloid cells and serves as the principal endogenous ligand for TLR4 and RAGE [20]. It regulates myeloid cell activation and accumulation and has been implicated in inflammatory and immunosuppressive responses driven by neutrophils and myeloid-derived suppressor cells (MDSCs), particularly their polymorphonuclear subset (PMN-MDSCs) [10,21,22]. Elevated S100A8/A9 expression has been reported across multiple solid tumors, including colorectal cancer, and correlates with MDSC accumulation, tumor progression, and poor clinical outcome [18,19,23]. In mouse models, genetic deletion of S100A9 reduces MDSC accumulation and suppresses both primary tumor growth and metastasis, including in the MC38 colon carcinoma model [24]. However, whether S100A9-associated myeloid inflammation is enhanced with aging and contributes to the growth of liver metastases remains largely unknown.

Here, we investigated how host aging alters immune cell composition in the liver and how these age-associated changes relate to the metastatic tumor microenvironment. We further examined whether modulation of the aged hepatic immune environment, through pharmacological targeting of the S100A8/A9 axis, alters the growth of liver metastases. Our goal was to clarify whether the aged hepatic immune environment represents not merely a bystander of tumor progression, but a functionally relevant context that can be modulated to influence metastatic outcome.

## 2. Materials and Methods

### 2.1. Animals and Cell Lines

Male C57BL/6 mice were used as young (8 weeks; CLEA Japan, Tokyo, Japan) and aged (>90 weeks; Oriental Yeast, Tokyo, Japan) cohorts. The age of the mice was selected to represent the advanced elderly population, based on established frailty scoring systems that correlate with biological aging in mice [25]. All mice were housed under standard specific pathogen-free conditions with ad libitum access to food and water. The murine colon carcinoma cell lines MC38 and its luciferase-expressing derivative (luci-MC38) were maintained as previously described [22]. All cells were confirmed to be free of mycoplasma contamination before use.

### 2.2. Liver Metastasis Model

Liver metastases were established by intrasplenic injection of 3 × 10^5^ MC38 or luci-MC38 cells under isoflurane anesthesia, followed by splenectomy to prevent primary splenic tumor growth as previously described [26]. Postoperative analgesia was provided with aspirin for three days. Successful tumor engraftment was confirmed by IVIS imaging two weeks post-inoculation. Detailed surgical procedures are described in the Supplementary Methods (Figure S1).

### 2.3. In Vivo Bioluminescence Imaging

In experiments using luciferase-expressing MC38 cells, tumor burden was monitored by in vivo bioluminescence imaging. Mice were injected intraperitoneally with D-luciferin (75 mg/kg; Cayman Chemical, Ann Arbor, MI, USA) and anesthetized with isoflurane. Ten minutes after luciferin administration, bioluminescence images were acquired using an IVIS Lumina LT imaging system (PerkinElmer, Waltham, MA, USA) with a 1 min exposure time under non-saturating, linear-range settings.

Images were analyzed using Living Image software, version4.4(PerkinElmer, Waltham, MA, USA), and tumor burden was quantified as radiance (photons/sec/cm^2^/sr). Background-subtracted radiance values were log_10_-transformed prior to statistical analysis.

### 2.4. Flow Cytometry

Single-cell suspensions were prepared from bone marrow, liver, and liver metastatic tumors. Bone marrow cells were flushed from femurs and tibias using phosphate-buffered saline (PBS). Liver and tumor tissues were minced and digested with collagenase, followed by filtration through a 70-μm cell strainer. Red blood cells were lysed using ammonium–chloride–potassium (ACK) lysis buffer.

Cells were stained with fluorochrome-conjugated antibodies (listed in Supplementary Table S1) following Fc receptor blockade. Data were acquired on a flow cytometer (BD Biosciences, San Jose, CA, USA) and analyzed using FlowJo software, version 10BD Biosciences, San Jose, CA, USA). Hematopoietic stem cells were defined as Lin^−^Sca-1^+^c-Kit^+^CD150^+^CD48^−^ cells. T cells were identified as CD3^+^ cells, with CD4^+^ and CD8^+^ subsets defined accordingly. Neutrophils were defined as CD11b^+^Ly6G^+^ cells, monocytes as CD11b^+^Ly6C^+^ cells, and macrophages as F4/80^+^ cells. Polymorphonuclear myeloid-derived suppressor cells (PMN-MDSCs) were defined as CD11b^+^Ly6G^+^Ly6Cint cells. To further distinguish them from conventional neutrophils, we incorporated CD244 as an additional marker (Ly6G^+^CD244^+^), consistent with previous reports characterizing the immunosuppressive phenotype of PMN-MDSCs in murine tumor models [8,9,10,22]. Representative gating strategies are shown in the Supplementary Materials (Figure S2).

### 2.5. RT–qPCR

Total RNA (1 µg) was isolated from liver tissues and reverse-transcribed into cDNA using the PrimeScript RT Reagent Kit with gDNA Eraser (Takara Bio Inc., Shiga, Japan). The resulting cDNA was diluted 50-fold, and 5 µL per reaction (equivalent to approximately 5 ng of RNA input) was used for quantitative PCR. Quantitative PCR was performed using TB Green Premix DimerEraser (Takara Bio Inc., Shiga, Japan) on a Thermal Cycler Dice Real Time System IV (Takara Bio Inc., Shiga, Japan). Cycling conditions consisted of 95 °C for 5 s and 60 °C for 45 s, repeated for 45 cycles. Gene expression levels were normalized to *Actb* (β-actin) as an internal control and calculated using the 2^−ΔΔCt^ method. Primer sequences used in this study are listed in Supplementary Table S2.

### 2.6. Western Blotting

Proteins were extracted from liver tissues or tumor samples using Cell Lysis Buffer (Nacalai Tesque, Inc., Kyoto, Japan) supplemented with Halt Protease and Phosphatase Inhibitor Cocktail (Thermo Fisher Scientific, Waltham, MA, USA). Protein concentrations were determined using a BCA assay. Equal amounts of protein (20 µg per lane) were separated by SDS–PAGE (8–15% gels) and transferred onto polyvinylidene fluoride (PVDF) membranes. Membranes were blocked with 5% bovine serum albumin and incubated with primary antibodies against S100A9 and β-actin (Supplementary Table S1). After incubation with horseradish peroxidase–conjugated secondary antibodies, signals were detected using enhanced chemiluminescence. Band intensities were quantified using ImageJ software (National Institutes of Health, Bethesda, MD, USA) and normalized to β-actin.

### 2.7. S100A9 Inhibition Study

S100A9 inhibition studies were performed using the small-molecule inhibitor Paquinimod (HY-100442; MedChemExpress, Monmouth Junction, NJ, USA), also known as ABR215757 [27,28]. The dose of 2 mg/kg was selected based on prior reports demonstrating effective S100A9 inhibition at comparable dose ranges in murine models [29], and was confirmed to be well tolerated in aged mice in preliminary experiments assessing general appearance, survival, and organ weights. Treatment commenced 4 weeks prior to tumor inoculation and continued until the endpoint at 21 days post-inoculation. Aged mice were randomly allocated into treatment groups (*n* = 10 per group), and tumor engraftment was confirmed by IVIS imaging at day 14. Only mice with detectable bioluminescence signals were included in subsequent analyses. The number of metastatic surface nodules was counted manually without formal blinding to treatment group allocation. The tumor area was quantified using ImageJ software, version 1.54g.

### 2.8. Serum Biochemistry and Histological Evaluation

Serum alanine aminotransferase (ALT) and aspartate aminotransferase (AST) levels were measured in tumor-naïve young and aged mice by an external laboratory (Kyudo Co., Ltd., Saga, Japan). For histological evaluation, liver tissues were fixed in 10% neutral-buffered formalin, embedded in paraffin, and sectioned at 4 µm. Sections were stained with hematoxylin and eosin (H&E) and Masson’s trichrome staining using standard protocols.

### 2.9. In Silico Analysis of Public Single-Cell RNA Sequencing (scRNA-Seq) Data

Publicly available scRNA-seq data of young and aged mouse livers were obtained from the Tabula Muris Senis consortium via the CELLxGENE portal [30], focusing on hepatocyte-annotated cell clusters.

Differential gene expression analysis was performed to identify age-associated changes in damage-associated molecular pattern (DAMP)-related genes. Data were visualized using volcano plots based on log2 fold change and adjusted *p*-values. For focused analysis, violin plots were generated to compare normalized *S100a9* expression levels across age groups.

### 2.10. Statistical Analysis

Statistical analyses were performed using GraphPad Prism software, version 10 (GraphPad Software, San Diego, CA, USA). Data are presented as mean ± standard error of the mean (SEM). Comparisons between two groups were conducted using Student’s *t*-test, paired *t*-test, or the Mann–Whitney U test when normality was not assumed. Pearson correlation coefficients were calculated for correlation analyses. A *p*-value of <0.05 was considered statistically significant.

## 3. Results

### 3.1. Aging Is Associated with Changes in Hepatic Immune Cell Composition in Tumor-Naïve Mice

**Figure 1:**
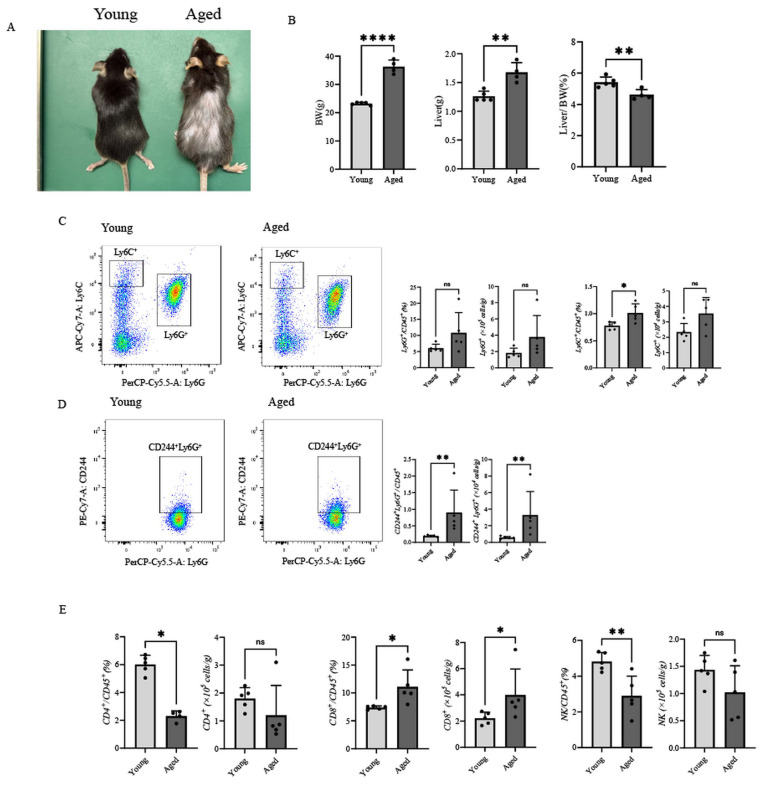
Age-associated alterations in hepatic immune composition in tumor-naïve mice. (**A**) Representative gross appearance of young (8-week-old) and aged (≥90-week-old) male C57BL/6 mice without tumor inoculation. (**B**) Body weight, liver weight, and liver weight–to–body weight ratio in young and aged mice (n = 5 young, n = 4 aged). (**C**–**E**) Flow cytometric profiling of liver immune cells, including Ly6G^+^ granulocytic cells, Ly6C^+^ monocytes, CD244^+^Ly6G^+^ cells, CD4^+^ T cells, CD8^+^ T cells, and NK cells. Absolute numbers are shown alongside frequencies (n = 5 per group, except for CD4^+^ T cells in aged mice where n = 4). Data are presented as mean ± SEM. Each dot in the bar graphs represents an individual mouse. Statistical analyses were performed using Student’s *t*-test or the Mann–Whitney U test, as appropriate. * *p* < 0.05, ** *p* < 0.01, **** *p* < 0.0001; ns, not significant.

To examine the effect of aging on hepatic immune cell composition before tumor inoculation, we analyzed tumor-naïve young (8-week-old) and aged (over 90-week-old) mice (Figure 1A). Body weight and liver weight were significantly increased in aged mice, whereas the liver-to-body weight ratio was significantly decreased compared with young mice (Figure 1B).

Flow cytometric analysis revealed clear differences in hepatic immune cell composition between young and aged mice (Figure 1C–E). In the myeloid compartment, the frequency of Ly6C^+^ monocytes was significantly increased in aged livers, whereas the proportion of Ly6G^+^ granulocytic cells was comparable between the two groups (Figure 1C). A substantial fraction of Ly6G^+^ cells expressed CD244, consistent with a PMN-MDSC-like phenotype, and the frequency of CD244^+^Ly6G^+^ cells was significantly increased in aged livers compared with young mice (Figure 1D). In the lymphoid compartment, the proportion of CD4^+^ T cells and NK cells was significantly lower in aged livers, whereas CD8^+^ T cells showed a modest but significant increase (Figure 1E). The absolute numbers of these immune cell populations were consistent in direction with the frequency data (Figure 1C–E). To assess whether baseline liver pathology may have contributed to the observed age-associated differences, serum ALT and AST levels and histological evaluation (H&E and Masson’s trichrome staining) were performed in tumor-naïve young and aged mice. No significant differences in serum ALT or AST levels were detected (Supplementary Figure S3), and histological analysis revealed no marked differences in hepatic inflammation or fibrosis between groups (Supplementary Figure S3). Together, these results show that aging is associated with measurable changes in hepatic immune cell composition in tumor-naïve mice, without overt baseline liver pathology. Additional baseline characterization data for tumor-naïve aged mice are shown in Figure S4.

### 3.2. Liver Metastases in Aged Hosts Exhibit a Myeloid-Dominant and Inflammatory Immune Microenvironment

**Figure 2:**
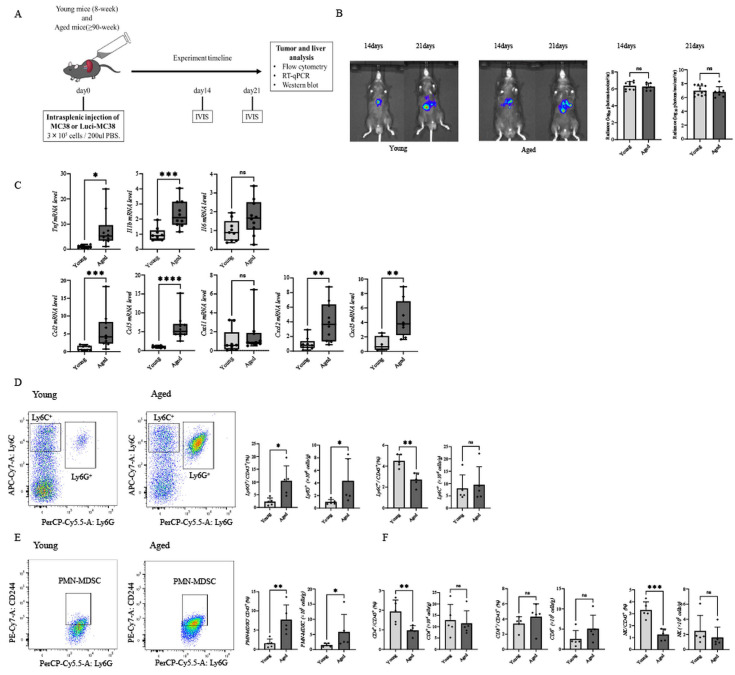
Age-associated alterations in hepatic immune cell composition in liver metastases characterized by Ly6G^+^ cell accumulation. (**A**) Schematic illustration of the experimental design for the liver metastasis model using intrasplenic injection of 3 × 10^5^ MC38 or luci-MC38 cells. (**B**) In vivo bioluminescence imaging (IVIS) of liver metastases in young and aged mice at days 14 and 21 after tumor inoculation, with representative images (left) and quantification of radiance (log_10_ photons/sec/cm^2/^sr) (right) (day 14: n = 9 young, n = 7 aged; day 21: n = 12 young, n = 7 aged). (**C**) Relative mRNA expression of pro-inflammatory factors (*Tnf*, *Il1b*, *Il6*, *Ccl2*, *Ccl5*, *Cxcl1*, *Cxcl2*, and *Cxcl5*) in metastatic livers at day 21 determined by RT–qPCR (n = 9 young and n = 10 aged mice, except for *Cxcl5* where n = 7 young and n = 8 aged mice). (**D**–**F**) Representative flow cytometry plots and frequencies of tumor-infiltrating immune cells at day 21, including Ly6G^+^ cells, Ly6C^+^ monocytes, polymorphonuclear myeloid-derived suppressor cells (PMN-MDSCs; Ly6G^+^CD244^+^), CD4^+^ and CD8^+^ T cells, and NK cells. Absolute numbers are shown alongside frequencies (n = 5 per group). Data are presented as mean ± SEM. Statistical analysis: Student’s *t*-test or Mann–Whitney U test. * *p* < 0.05, ** *p* < 0.01, *** *p* < 0.001, **** *p* < 0.0001; ns, not significant.

To examine how aging influences the immune contexture of liver metastases, we first established a syngeneic liver metastasis model by intrasplenic injection of MC38 or luci-MC38 cells into young and aged mice (Figure 2A). Longitudinal in vivo bioluminescence imaging (IVIS) performed at days 14 and 21 after tumor inoculation showed progressive metastatic growth in both groups. There was no significant difference in the overall metastatic tumor burden, as quantified by radiance, between young and aged hosts at either time point (Figure 2B). These results indicate that aging does not simply accelerate macroscopic tumor growth in this liver metastasis model.

RT–qPCR analysis revealed that metastatic livers from aged mice showed increased expression of multiple inflammatory factors, including *Tnf*, *Il1b*, *Ccl2*, *Ccl5*, *Cxcl2*, and *Cxcl5*, whereas *Il6* and *Cxcl1* levels were not significantly different between the two groups (Figure 2C).

Flow cytometric analysis revealed a difference in the composition of the tumor-infiltrating immune cell compartment in aged mice (Figure 2D–F). Within the myeloid compartment, metastatic lesions in aged hosts showed a significant increase in Ly6G^+^ granulocytic cells, concomitant with a significant reduction in the frequency of Ly6C^+^ monocytes (Figure 2D). In addition, the frequency of PMN-MDSCs was significantly increased in the tumors of aged mice (Figure 2E). In the lymphoid compartment, the frequencies of CD4^+^ T cells and NK cells were significantly reduced in aged mice, whereas the frequency of CD8^+^ T cells was not significantly decreased (Figure 2F). The absolute numbers of these populations were consistent with the frequency data (Figure 2D–F).

Together, these results indicate that, although aging does not increase the overall burden of liver metastases in this model, it is associated with a myeloid-dominant and inflammatory immune cell composition within metastatic lesions.

### 3.3. S100A9 Is Selectively Upregulated in the Aged Liver and Liver Metastases

**Figure 3:**
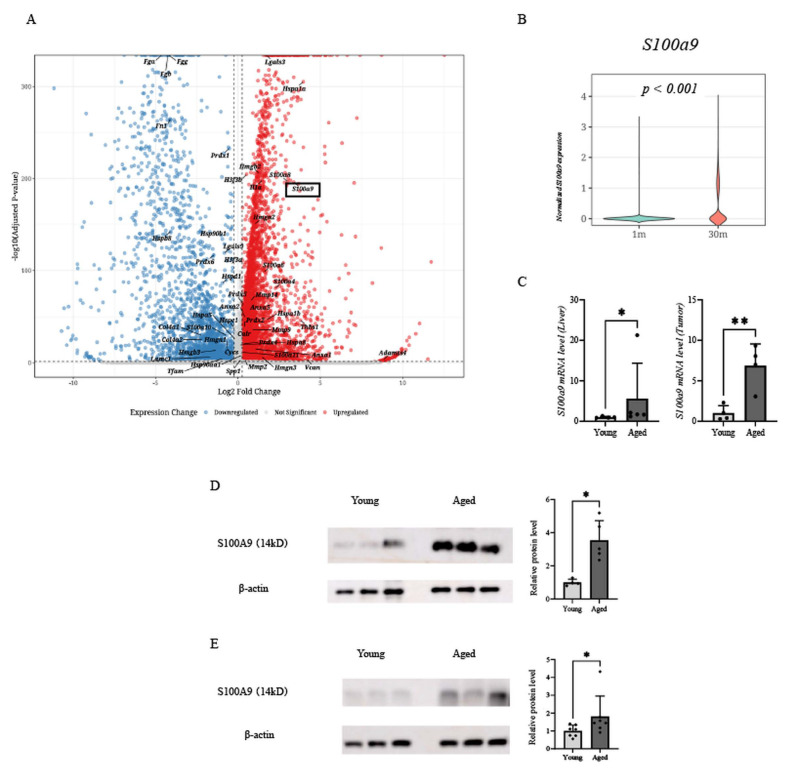
Higher expression of S100A9 in aged livers and metastatic lesions. (**A**) Volcano plot of differentially expressed genes in aged compared with young livers using the Tabula Muris Senis database. Red and blue dots represent significantly upregulated and downregulated genes in aged tissues, respectively (criteria: |log_2_ fold change| > 1.0 and *p* < 0.05). *S100a9* is identified as one of the significantly upregulated genes. (**B**) Normalized *S100a9* expression levels in young and aged livers from the same dataset. (**C**) Relative *S100a9* mRNA expression in tumor-naïve livers and metastatic tumors from young and aged mice. Expression levels were normalized to *Actb* (β-actin) (Liver: n = 4 young, n = 5 aged; Tumor: n = 4 per group). (**D**,**E**) Western blot analysis of S100A9 protein levels in (**D**) tumor-naïve livers and (**E**) metastatic tumors. Representative blots (left) and quantification of S100A9 protein levels (right) are shown. Protein levels were normalized to β-actin (Liver: n = 4 young, n = 5 aged; Tumor: n = 7 per group). Data are presented as mean ± SEM of at least three independent experiments. Statistical analyses were performed using Student’s *t*-test or the Mann–Whitney U test, as appropriate. * *p* < 0.05, ** *p* < 0.01. The whole blots (uncropped blots) are shown in Supplementary file.

To identify candidates for inflammatory mediators associated with aging in the liver, we performed an in silico analysis using public single-cell RNA-seq data from the Tabula Muris Senis consortium, focusing on hepatocyte-annotated cell clusters. Differential expression analysis identified *S100a9* as one of the highly upregulated genes in hepatocyte-annotated clusters in the aged liver dataset (Figure 3A). Consistently, analysis within the same cohort showed that normalized *S100a9* expression levels were significantly higher in aged livers than in young controls (Figure 3B). Parallel in silico analyses of *S100a8*, a known binding partner of *S100a9*, are presented in Supplementary Figure S5.

We next validated these bioinformatic findings using our experimental model. RT–qPCR analysis confirmed that *S100a9* mRNA levels were significantly higher in both the tumor-naïve livers and metastatic tumors of aged mice (Figure 3C). Western blot analysis further showed that S100A9 protein expression was significantly elevated in both the aged tumor-naïve liver (Figure 3D) and the metastatic tumor tissues (Figure 3E).

Together, these findings indicate coordinated upregulation of the S100A8/A9 axis in both the aged liver and metastatic lesions.

### 3.4. Pharmacological Modulation of the S100A8/A9 Axis Significantly Reduces the Growth of Liver Metastases in Aged Hosts

**Figure 4:**
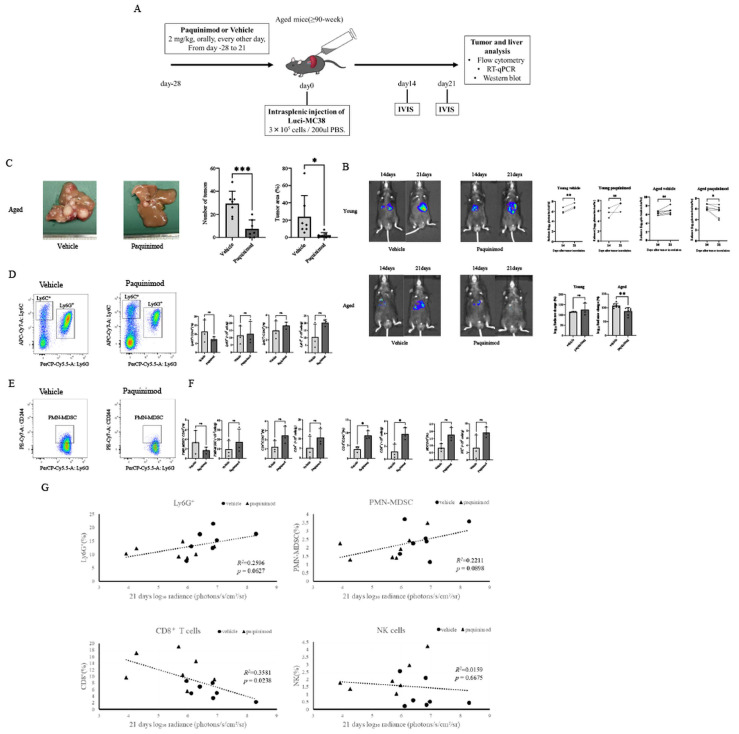
Association of S100A9 inhibition with lower tumor burden and altered immune cell composition in aged metastatic livers. (**A**) Experimental schema of tumor cell inoculation and every other day paquinimod or vehicle administration in young and aged mice. (**B**) Representative IVIS bioluminescence images of liver metastases at days 14 and 21 after tumor inoculation (left). Comparisons of tumor burden (radiance) between day 14 and day 21 within each treatment group are shown (upper right); paired *t*-tests were performed (Young: n = 3 per group; Aged: n = 7 per group). The change in tumor burden (Δlog10 photons/sec/cm^2^/sr) from day 14 to day 21 is summarized (lower right) for young and aged mice. For the Δlog10 bar graphs, vehicle- and paquinimod-treated mice were compared using unpaired Student’s *t*-tests (n = 3 young and n = 7 aged). (**C**) Representative macroscopic appearance of livers (left) and quantification of the number of metastatic nodules and the tumor area ratio (right) at day 21 (n = 7 per group). (**D**–**F**) Representative flow cytometry plots and frequencies of tumor-infiltrating immune cells at day 21 after tumor inoculation, including Ly6G^+^ cells, Ly6C^+^ monocytes, PMN-MDSCs (Ly6G^+^CD244^+^), CD4^+^ and CD8^+^ T cells, and NK cells. Absolute numbers are shown alongside frequencies (n = 3 per group). (**G**) Correlation between day 21 tumor burden and frequencies of the indicated immune cell populations in aged mice, assessed by simple linear regression; *R*^2^ and *p* values are shown. (n = 14 aged mice). Data are presented as mean ± SEM. Statistical analyses were performed using Student’s *t*-test, paired *t*-test, or the Mann–Whitney U test, as appropriate. * *p* < 0.05, ** *p* < 0.01, *** *p* < 0.001.

To examine whether modulation of the aged hepatic immune environment affects metastatic outcome, we treated young and aged mice with paquinimod, a small-molecule inhibitor of the S100A8/A9 axis. Treatment was initiated four weeks prior to tumor inoculation to target the pre-established immune microenvironment rather than the tumor itself (Figure 4A). Longitudinal IVIS imaging at days 14 and 21 after tumor inoculation showed no significant difference in tumor-associated radiance in young mice at either time point (*p* = 0.5998). In contrast, in aged mice, paquinimod-treated animals showed a smaller increase in bioluminescent signals between days 14 and 21, resulting in a significantly lower tumor burden at day 21 compared with vehicle-treated controls (Figure 4B).

These imaging findings were consistent with macroscopic analysis at day 21, which showed that paquinimod-treated aged mice had a significantly lower metastatic burden, as assessed by both the number of surface tumor nodules and the total tumor area ratio (Figure 4C).

To examine the immune cell composition of the metastatic lesions, flow cytometric analysis of tumor-infiltrating immune cells was performed using a unified panel and gating strategy (Figure 4D–F). In the myeloid compartment, Ly6C/Ly6G profiling showed a trend toward reduced frequencies of Ly6G^+^ cells in paquinimod-treated aged mice (Figure 4D). Consistently, further gating demonstrated a decreasing trend in PMN-MDSCs within the metastatic liver following paquinimod treatment (Figure 4E).

In parallel, analysis of the lymphoid compartment showed increased frequencies of CD8^+^ T cells, whereas CD4^+^ T cells and NK cells showed no significant changes (Figure 4F). The absolute numbers of these populations were consistent in direction with the frequency data (Figure 4D–F).

Finally, Pearson correlation and linear regression analysis in aged mice revealed that tumor burden tended to correlate positively with the frequencies of Ly6G^+^ cells (*R*^2^ = 0.2596, *p* = 0.0627) and PMN-MDSCs (*R*^2^ = 0.2211, *p* = 0.0898), although these associations did not reach statistical significance. In contrast, tumor burden showed a significant inverse correlation with the frequency of tumor-infiltrating CD8^+^ T cells (*R*^2^ = 0.3581, *p* = 0.0238), whereas no such correlation was observed for NK cells (*R*^2^ = 0.0159, *p* = 0.6675; Figure 4G).

Collectively, these findings suggest that pharmacological modulation of the S100A8/A9 axis is associated with reduced growth of liver metastases in aged mice and is accompanied by changes in the immune cell composition of the metastatic lesions.

## 4. Discussion

Aging profoundly influences tumor immunity and therapeutic responsiveness. In this study, we show that aging is associated with a distinct shift in hepatic immune cell composition that is already evident under tumor-naïve conditions and persists during liver metastasis. In tumor-naïve livers, aged hosts exhibit a myeloid-skewed immune composition, and liver metastases developing in these hosts display a myeloid-rich, inflammatory immune environment. We further identify S100A9 as a molecule selectively upregulated in the aged liver and demonstrate that pharmacological modulation of the S100A8/A9 axis significantly reduces the growth of liver metastases in aged, but not young, hosts. These observations indicate that, in the aged liver, the S100A8/A9 axis is not merely a correlative feature but becomes functionally relevant to the growth of liver metastases.

In this study, we compared tumor-naïve livers and metastatic lesions in the same hosts and found that aged mice exhibit a myeloid-skewed immune profile in the tumor-naïve liver [7,31], whereas metastatic lesions display a distinct Ly6G^+^/PMN-MDSC–biased inflammatory immune profile [8,32]. In this context, the S100A8/A9 axis is widely recognized as a central molecular program that defines neutrophil- and PMN-MDSC–driven inflammatory and immunosuppressive myeloid responses [10,20,22]. Consistent with this conceptual framework, S100A9 was already elevated in the tumor-naïve liver and remained high in metastatic lesions, thereby placing our findings within an S100A8/A9-defined established myeloid immune context of the aged liver [16,17]. Previous mouse studies have established a functional requirement for S100A9 in tumor progression: S100A9-deficient mice exhibit markedly reduced MDSC accumulation and suppressed growth and metastasis of MC38 and other syngeneic tumors [24,33]. These observations position S100A9 as not merely a marker of inflammation but as an active driver of the myeloid immune context in which tumors develop.

Paquinimod significantly reduces the growth of liver metastases in aged, but not young, hosts. This difference in therapeutic response is consistent with the markedly higher baseline expression of S100A9 in the aged liver, and supports the interpretation that the functional relevance of the S100A8/A9 axis is dependent on the immune context defined by elevated S100A9 expression. S100A9 has been shown to promote MDSC recruitment and activation through TLR4- and RAGE-dependent signaling, and its inhibition operates primarily through modulation of myeloid cell function rather than through direct effects on tumor cells [20,23]. Consistent with this, paquinimod did not affect cell viability or NF-κB activity in MC38 cells in vitro (Supplementary Figure S6), indicating that the observed therapeutic effect is mediated through immune modulation rather than direct effects on tumor cells. These findings raise the possibility that modulation of the myeloid compartment by paquinimod may be associated with greater CD8^+^ T cell frequency within metastatic lesions, although whether this reflects enhanced antitumor activity remains to be determined. We selected Ly6G^+^ cells, PMN-MDSCs, CD8^+^ T cells, and NK cells for correlation analysis because they showed the most prominent and treatment-responsive changes in the preceding flow cytometric analyses. Although paquinimod primarily targets the myeloid compartment [27,28], our correlation analysis indicates that tumor burden is more closely associated with CD8^+^ T cell infiltration than with PMN-MDSCs. This implies that modulation of the myeloid compartment by paquinimod may contribute to tumor control indirectly, at least in part, by permitting more effective CD8^+^ T cell–mediated antitumor responses [10,15,22].

Direct in vivo evidence of S100A8/A9 pathway engagement following paquinimod treatment was not obtained in the present study. Assessment of S100A9 protein levels by Western blotting in tumor-free aged mice following paquinimod treatment revealed no statistically significant change (Supplementary Figure S7). Although a trend toward reduced expression of inflammatory cytokines (*Tnf* and *Il6*) was observed in metastatic livers of aged mice following paquinimod treatment (Supplementary Figure S8), these changes did not reach statistical significance. The precise mechanisms through which paquinimod modulates the immune microenvironment in aged hosts, therefore, remain to be elucidated.

From a translational perspective, these findings highlight the importance of considering host age and tissue context in the treatment of liver metastasis [2,14]. Rather than applying uniform therapeutic strategies across all patients, approaches tailored to the immune context of the aged liver may be particularly relevant for elderly patients [4,34]. In this regard, paquinimod has already been evaluated in clinical settings [27], supporting the feasibility of targeting the S100A8/A9 axis in patients. Although our model does not reproduce the chronic course of metastatic disease, the use of identical tumor cells in young and aged hosts allows isolation of the contribution of the host immune environment to metastatic growth. Whether this approach is also effective as a therapeutic intervention after tumor establishment remains to be investigated.

This study has several limitations. The experimental model was constrained to male C57BL/6 mice, a single tumor cell line (MC38), and a fixed 21-day observation window; aged mice (>90 weeks) represent a scarce resource with high biological variability, which restricted cohort sizes in some experiments. Generalizability to female hosts, other tumor types, other genetic backgrounds, and the chronic course of metastatic progression in elderly patients, therefore, remains to be addressed. In addition, functional assessment of CD8^+^ T cells, direct in vivo evidence of S100A8/A9 target engagement, and age-related pharmacokinetic differences were not evaluated in the present study. Nevertheless, we believe that the present study provides a useful experimental framework for investigating age-dependent host factors in liver metastasis.

## 5. Conclusions

In conclusion, we demonstrate that aging is associated with a myeloid-biased hepatic immune environment and markedly elevated hepatic S100A9 expression, creating a distinct immune context that is functionally relevant to liver metastasis growth. Pharmacological modulation of the S100A8/A9 axis with paquinimod selectively reduced metastatic burden in aged, but not young, hosts, accompanied by an increased representation of CD8^+^ T cells in the metastatic lesions. These findings position the S100A8/A9 axis as a context-specific therapeutic target for liver metastasis in aged hosts.

## Data Availability

The data presented in this study are available from the corresponding author upon reasonable request.

## Supplementary Materials

The following supporting information can be downloaded at: https://www.mdpi.com/article/10.3390/cancers18101635/s1, Figure S1: Schematic illustration of the intrasplenic injection model for liver metastasis; Figure S2: Gating strategy; Figure S3: Baseline liver pathology in tumor-naïve young and aged mice; Figure S4: Aging induces myeloid-biased hematopoiesis and hepatic senescence marker upregulation in tumor-naïve mice; Figure S5: Higher expression of *S100a8* in aged livers and metastatic lesions; Figure S6: Paquinimod does not affect viability or NF-κB activity in MC38 cells; Figure S7: S100A9 protein levels in tumor-free aged mice following paquinimod treatment; Figure S8: Inflammatory cytokine expression in metastatic livers of aged mice following paquinimod treatment; Table S1: Antibodies used in this study; Table S2: Primer sequences used for RT–qPCR.

## Abbreviations

The following abbreviations are used in this manuscript:

DAMP	Damage-associated molecular pattern
IVIS	In vivo bioluminescence imaging
MDSC	Myeloid-derived suppressor cell
PMN-MDSC	Polymorphonuclear myeloid-derived suppressor cell
RT–qPCR	Quantitative reverse transcription PCR
TME	Tumor microenvironment
