# Supplementary material for: Paquinimod Targeting of the S100A8/A9 Axis Suppresses Liver Metastasis in Aged Mice
Source: Cancers (Basel). 2026 May 19;18(10):1635. doi: 10.3390/cancers18101635 (PMC13204323; doi:10.3390/cancers18101635)
Supplement: Supplementary file 1 [file cancers-18-01635-s001.zip › cancers - 4294484 Supplementary Materials.pdf]

Figure S1

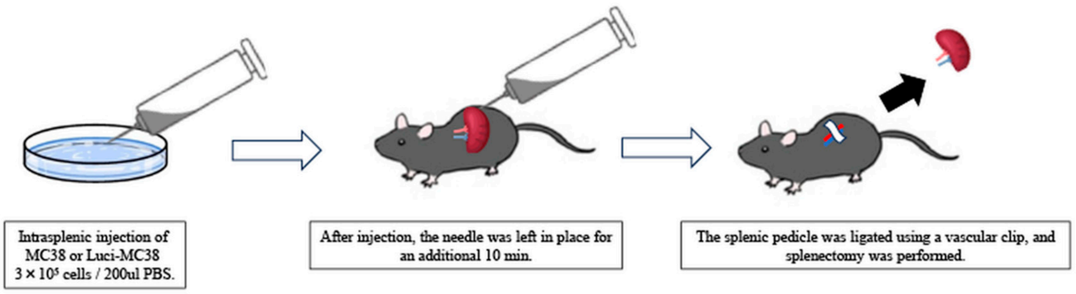

Supplementary Figure S1. Schematic illustration of the intrasplenic injection model for liver metastasis.

MC38 or luciferase-expressing MC38 (luci-MC38) cells were injected into the spleen under inhalational anesthesia to establish a liver metastasis model. Tumor cells (3×10^5 cells in 200 μL PBS) were slowly injected into the upper pole of the spleen parenchyma using a 26-gauge needle over approximately 2 min. To prevent leakage of the cell suspension and subsequent peritoneal seeding, the needle was left in place for an additional 10 min before withdrawal. Splenectomy was subsequently performed by ligation of the splenic pedicle to prevent primary tumor growth in the spleen. The abdominal cavity was rinsed with PBS if cell spillage was observed, followed by closure of the abdominal wall and skin. Tumor growth was confirmed by IVIS imaging at day 14 after tumor inoculation.

Figure S2

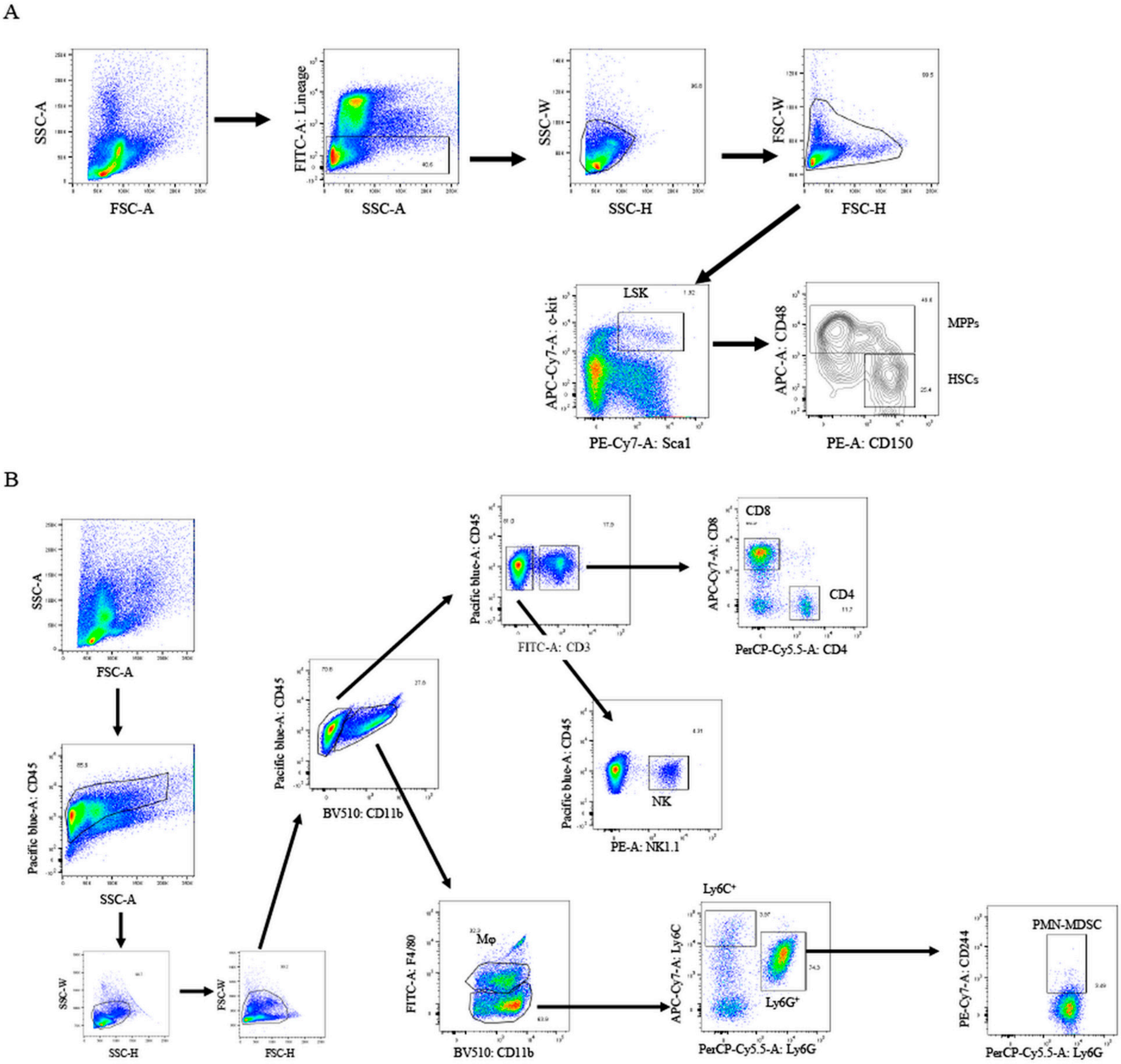

Supplementary Figure S2. Gating strategy

A (Bone marrow).

Sequential gating for hematopoietic stem and progenitor cells (HSPCs): after coarse debris/doublet exclusion, Lineage^{-} cells were gated, followed by identification of LSK (Lin^{-}Sca-1^{+}c-Kit^{+}) and subdivision into hematopoietic stem cells (HSCs) (CD150^{+}CD48^{-}) and multipotent progenitor cells (MPPs) (CD150^{-}CD48^{+}).

B (Liver/Tumor).

After exclusion of debris (FSC-A vs. SSC-A) and doublets (SSC-W vs. SSC-H and FSC-W vs. FSC-H), CD45^{+} leukocytes were gated. CD45^{+} cells were subsequently subdivided into CD3^{+} or NK1.1^{+}

lymphoid and $CD11b^+$ myeloid compartments. T cells ($CD3^+$) were classified into $CD4^+$ and $CD8^+$ subsets. NK cells were identified as $CD45^+NK1.1^+$ cells. Within the myeloid compartment, macrophages were identified as $CD11b^+F4/80^+$ cells. Myeloid-derived suppressor cells (MDSCs) were subdivided into monocytic MDSCs (M-MDSCs; $CD11b^+Ly6G^-Ly6C^{high}$), and polymorphonuclear MDSCs (PMN-MDSCs; $CD11b^+Ly6G^+Ly6C^{int}$). PMN-MDSCs were distinguished from neutrophils based on CD244 expression. Identical gating strategies were applied to both tumor-naive livers and metastatic tumor tissues.

Figure S3

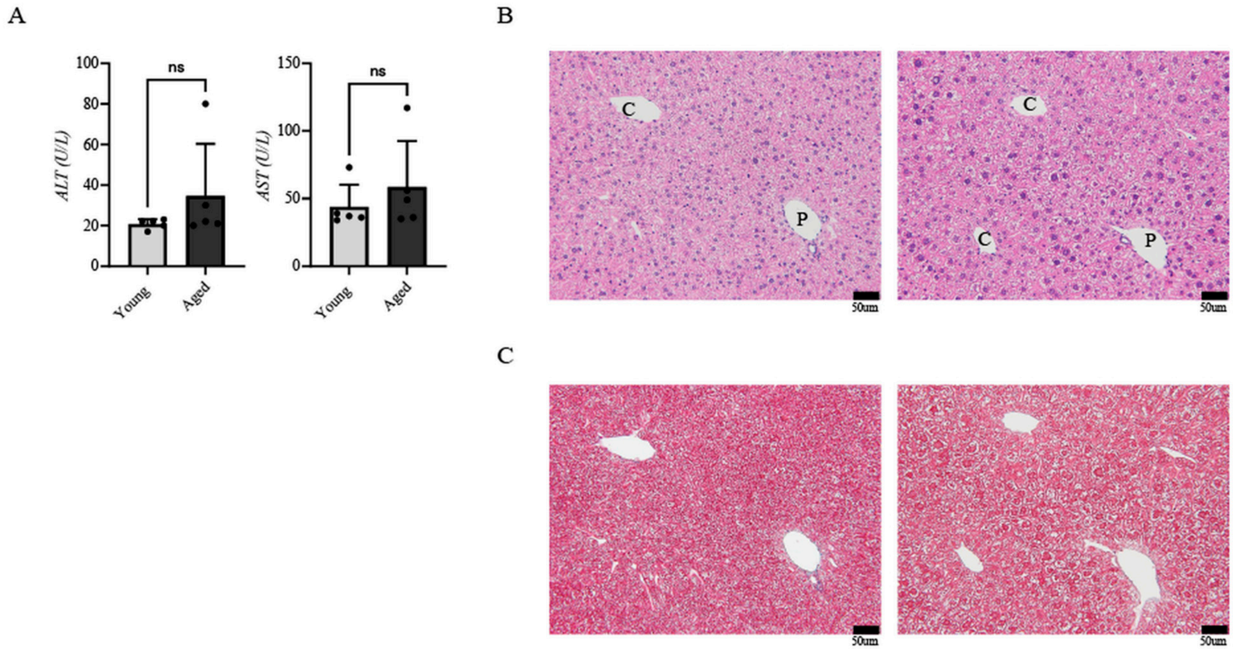

Supplementary Figure S3. Baseline liver pathology in tumor-naïve young and aged mice.

(A) Serum alanine aminotransferase (ALT) and aspartate aminotransferase (AST) levels in tumor-naïve young (8-week-old) and aged (≥90-week-old) male C57BL/6 mice (n = 5 per group).

(B) Representative hematoxylin and eosin (H&E)-stained liver sections from young and aged tumor-naïve mice. Scale bar: 50 µm.

(C) Representative Masson's trichrome-stained liver sections from young and aged tumor-naïve mice. Scale bar: 50 µm.

Data are presented as mean ± SEM. Statistical analyses were performed using Student's t-test or the Mann–Whitney U test, as appropriate. ns, not significant.

Figure S4

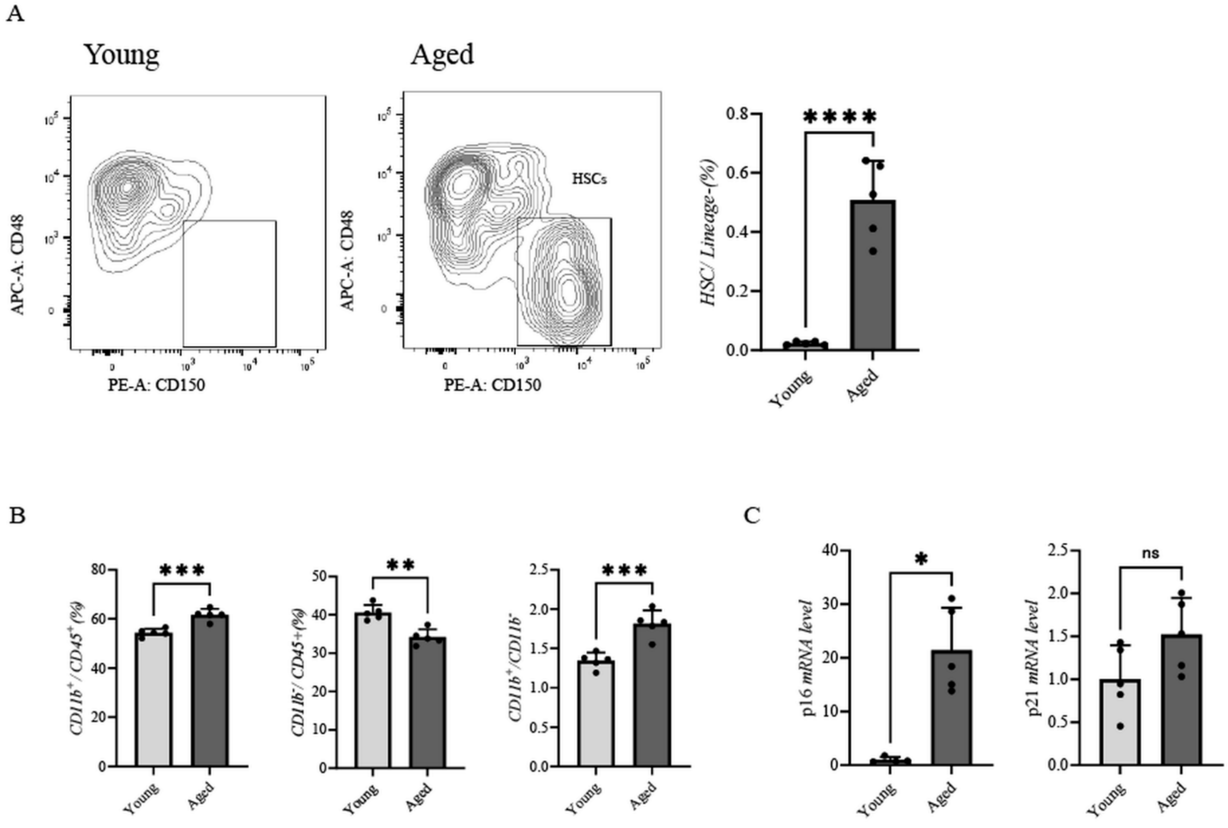

Supplementary Figure S4. Aging induces myeloid-biased hematopoiesis and hepatic senescence marker upregulation in tumor-naïve mice.

(A) Flow cytometric analysis of hematopoietic stem cells (HSCs; $Lin^{-}Sca\text{-}1^{+}c\text{-}Kit^{+}CD150^{+}CD48^{-}$) in the bone marrow of young (8-week-old) and aged (≥90-week-old) male C57BL/6 mice, including representative plots (left) and quantification of HSC frequency (right) ($n = 5$ per group).

(B) Increased frequency of $CD11b^{+}$ myeloid cells in the bone marrow of aged mice. Representative plots (left) and quantification of $CD11b^{+}$ and $CD11b^{-}$ populations and the $CD11b^{+}/CD11b^{-}$ ratio (right) are shown ($n = 5$ per group).

(C) Relative mRNA expression levels of senescence-associated markers p16 (encoded by *Cdkn2a*) and p21 (encoded by *Cdkn1a*) in livers from young (8-week-old) and aged (≥90-week-old) male C57BL/6 mice, as determined by RT–qPCR. Expression levels were normalized to *Actb* (β-actin) and are presented relative to the young group.

For p16, $n = 4$ young and $n = 5$ aged; for p21, $n = 5$ per group.

Data are presented as mean ± SEM. Statistical analyses were performed using Student's t-test or the Mann–Whitney U test, as appropriate. * $p < 0.05$, ** $p < 0.01$, *** $p < 0.001$; ns, not significant.

Figure S5

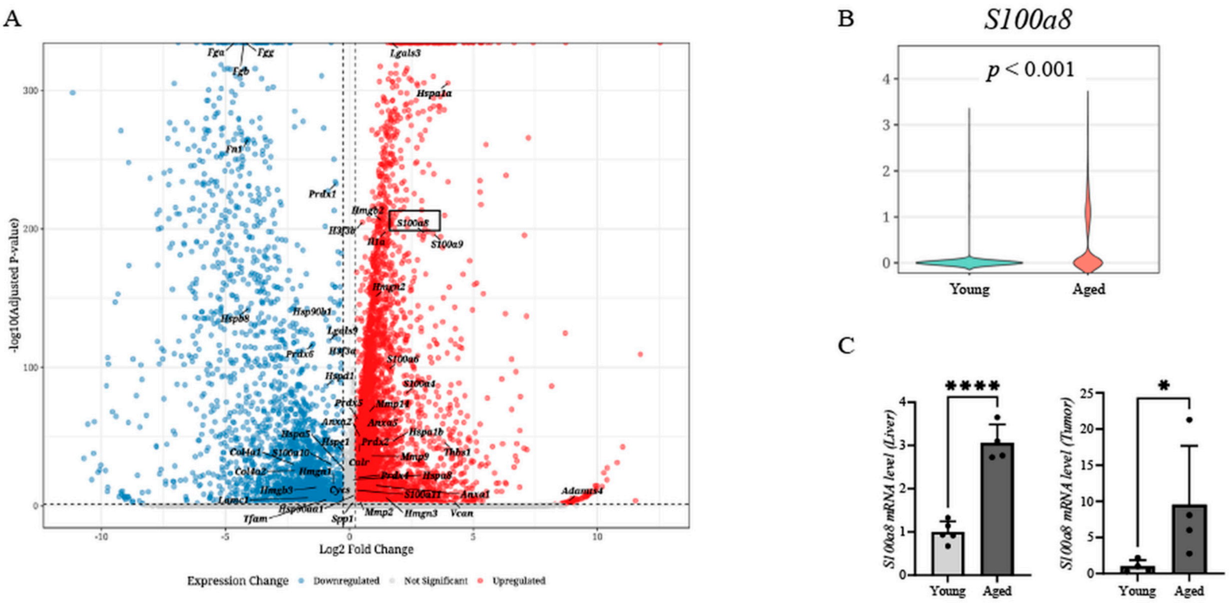

Supplementary Figure S5. Higher expression of *S100a8* in aged livers and metastatic lesions.
(A) Volcano plot of differentially expressed genes in aged compared with young livers using the Tabula Muris Senis database. Red and blue dots represent significantly upregulated and downregulated genes in aged tissues, respectively (criteria: $|\log_2 \text{fold change}| > 1.0$ and $p < 0.05$). *S100a8* is identified as one of the significantly upregulated genes.
(B) Normalized *S100a8* expression levels in young and aged livers from the same dataset.
(C) Relative *S100a8* mRNA expression in tumor-naïve livers and metastatic tumors from young and aged mice. Expression levels were normalized to *Actb* (β-actin) (Liver: $n = 5$ young, $n = 4$ aged; Tumor: $n = 4$ per group).
Data are presented as mean ± SEM of at least three independent experiments. Statistical analyses were performed using Student's t-test or the Mann–Whitney U test, as appropriate. * $p < 0.05$, ** $p < 0.01$, *** $p < 0.001$.

Figure S6

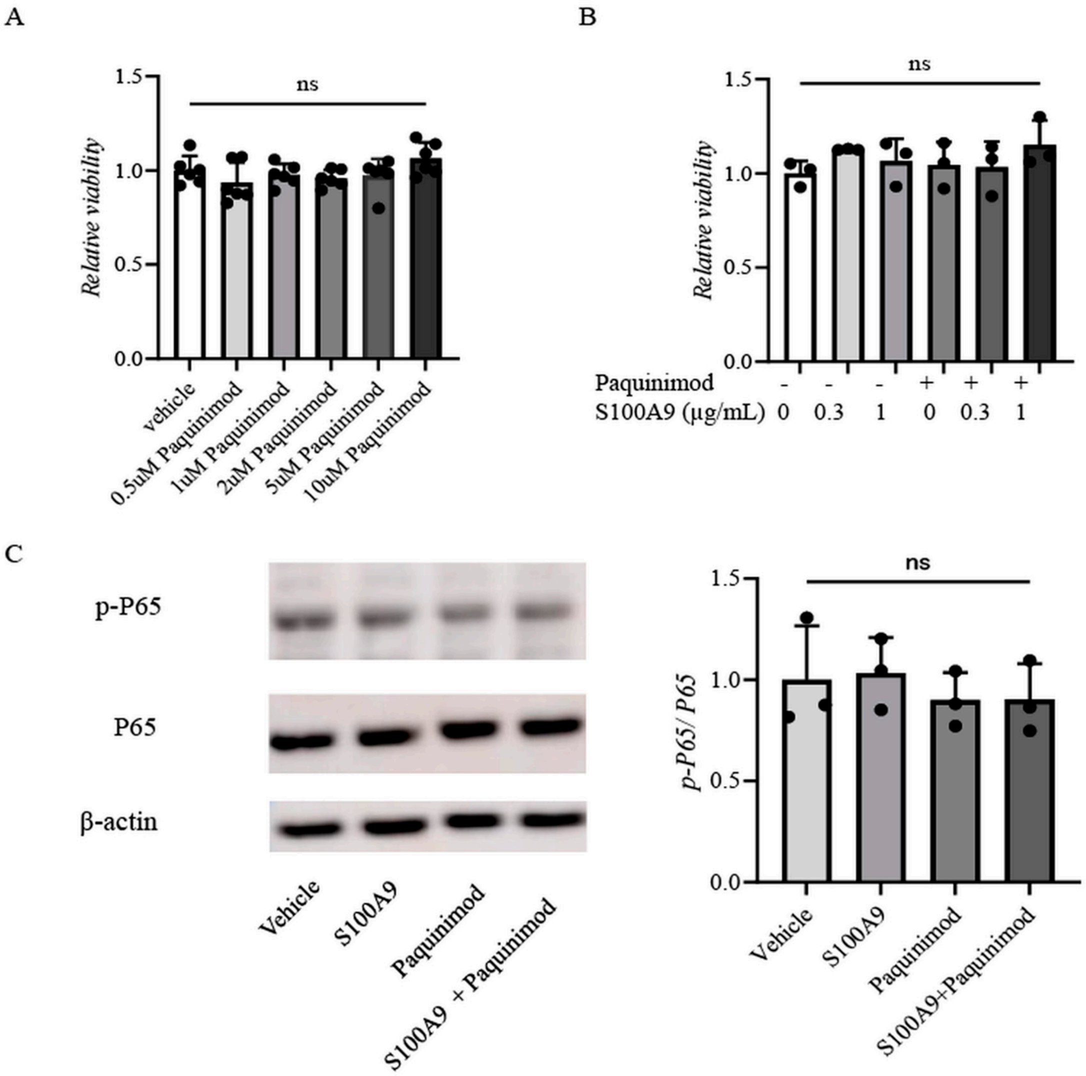

Supplementary Figure S6. Paquinimod does not affect viability or NF-κB activity in MC38 cells *in vitro*.

(A) Cell viability of MC38 cells treated with paquinimod at concentrations of 0.5, 1, 2, 5, and 10 μM for 24 hours, assessed by WST-8 assay ($n = 6$).

(B) Cell viability of MC38 cells treated with or without paquinimod (2 μM) in the presence of recombinant S100A9 (0, 0.3, or 1 μg/mL) for 24 hours, assessed by WST-8 assay ($n = 6$).

(C) Western blot analysis of phosphorylated NF-κB p65 (p-P65) and total P65 in MC38 cells treated with vehicle, recombinant S100A9 (1 μg/mL), paquinimod (2 μM), or S100A9 combined with paquinimod for 24 hours. β-Actin was used as a loading control. Quantification of the p-P65/P65 ratio is shown (right) ($n = 3$).

Data are presented as mean ± SEM. ns, not significant.

Figure S7

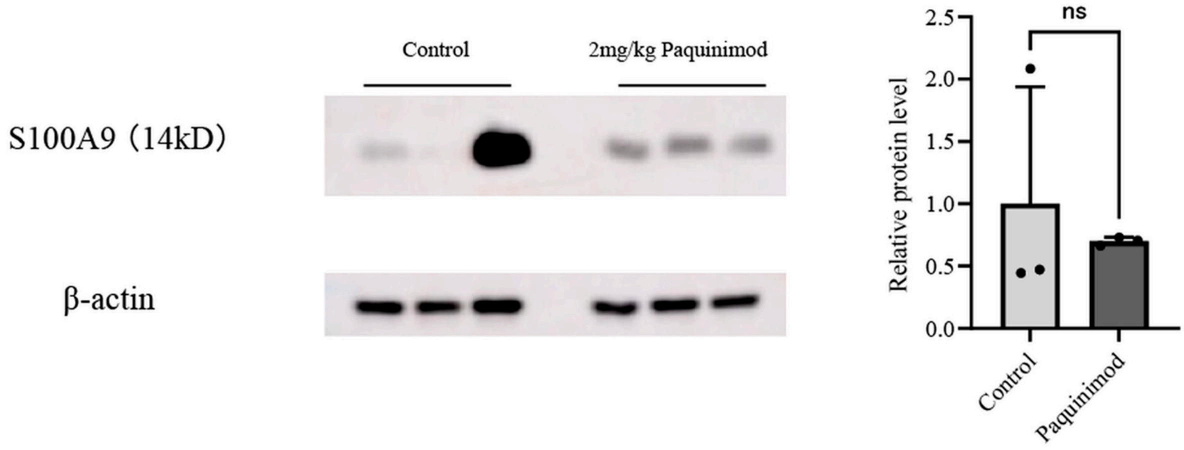

Supplementary Figure S7. S100A9 protein levels in tumor-free aged mice following paquinimod treatment.

Representative Western blot images (left) and quantification of S100A9 protein levels (right) in tumor-free liver tissues from aged mice treated with vehicle or paquinimod for 4 weeks. Protein levels were normalized to β-actin (*n* = 3 per group).

Data are presented as mean ± SEM. Statistical analyses were performed using Student's t-test or the Mann–Whitney U test, as appropriate. ns, not significant.

Figure S8

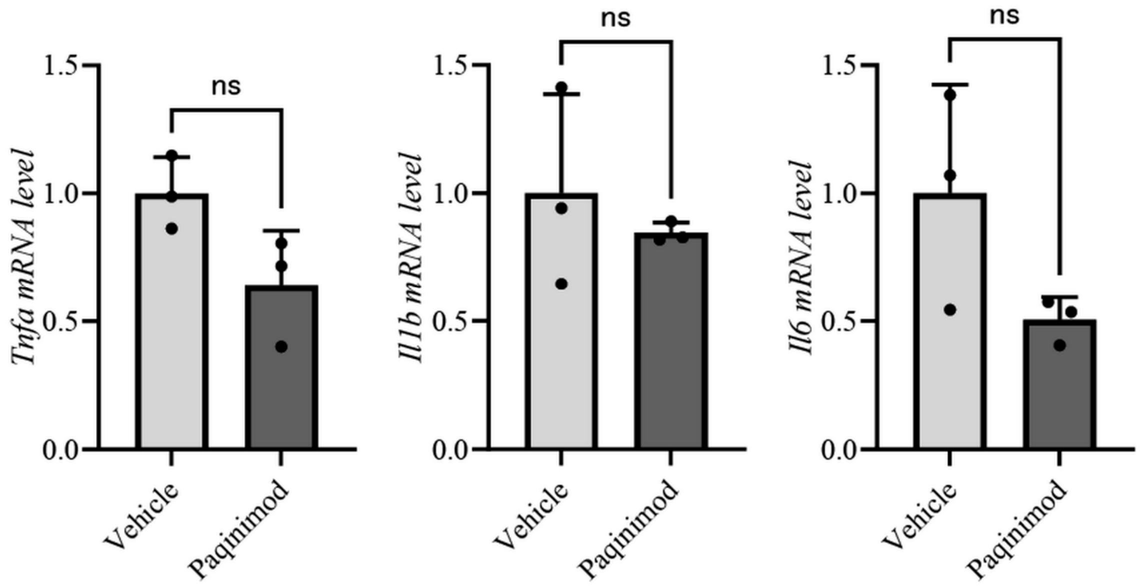

Supplementary Figure S8. Inflammatory cytokine expression in metastatic livers of aged mice following paquinimod treatment.

Relative mRNA expression levels of *Tnf*, *Il1b*, and *Il6* in metastatic liver tissues from aged mice treated with vehicle or paquinimod, determined by RT–qPCR. Expression levels were normalized to *Actb* (β-actin) (*n* = 3 per group).

Data are presented as mean ± SEM. Statistical analyses were performed using Student's t-test or the Mann–Whitney U test, as appropriate. ns, not significant.

Supplementary Table S1. Antibodies used in this study

Target	Application	Clone	Host species	Conjugate	Manufacturer	Catalog No.	Dilution
CD45	Flow cytometry	104	Mouse	Pacific Blue	BioLegend	#109820	1:100
CD11b	Flow cytometry	M1/70	Rat	BV510	BioLegend	#101263	1:100
CD3	Flow cytometry	17A2	Rat	FITC	BioLegend	#100204	1:100
CD4	Flow cytometry	GK1.5	Rat	PerCP5.5	BioLegend	#100434	1:100
CD8	Flow cytometry	53-6.7	Rat	APC/Cy7	BioLegend	#100714	1:100
PD-1	Flow cytometry	RMP1-30	Rat	PE/Cy7	BioLegend	#109109	1:100
NK1.1	Flow cytometry	S17016D	Mouse	PE	BioLegend	#156504	1:100
F4/80	Flow cytometry	BM8	Rat	FITC	BioLegend	#123107	1:100
Ly6G	Flow cytometry	1A8	Rat	PerCP5.5	BioLegend	#127615	1:100
Ly6C	Flow cytometry	HK1.4	Rat	APC/Cy7	BioLegend	#128025	1:100
CD244	Flow cytometry	m2B4	Mouse	PE/Cy7	BioLegend	#133511	1:100
Lin	Flow cytometry	145-2C11, RB6-8C5, RA3-6B2, Ter-119, M1/70	Rat/Hamster	FITC	BioLegend	#133301	1:100
Sca-1	Flow cytometry	D7	Rat	PE/Cy7	BioLegend	#108114	1:100
c-Kit	Flow cytometry	2B8	Rat	APC/Cy7	BioLegend	#105825	1:100
CD150	Flow cytometry	TC15-12F12.2	Rat	PE	BioLegend	#162605	1:100
CD48	Flow cytometry	HM48-1	Armenian Hamster	APC	BioLegend	#103411	1:100
FcR Blocking Reagent	Flow cytometry	-	-	-	Miltenyi Biotec B.V. & Co. KG	#130-092-575	as instructed

Target	Application	Clone	Host species	Conjugate	Manufacturer	Catalog No.	Dilution
S100A9	Western blot	D3U8M	Rabbit	-	Cell Signaling	#73425S	1:1000
NF-kappaB p65	Western blot	D14E12	Rabbit		Cell Signaling	#8242	1:1000
Phospho-NF-kappaB p65 (Ser536)	Western blot	93H1	Rabbit		Cell Signaling	#3033	1:1000
β-Actin	Western blot	13E5	Rabbit	-	Cell Signaling	#4970S	1:1000
HRP-conjugated	Western blot	polyclonal	Goat	-	Proteintech	#SA00001-2	1:10000

Supplementary Table S2. Primer sequences used for RT–qPCR

Gene	Forward (5’→3’)	Reverse (5’→3’)
p16 (*Cdkn2a*)	CCCAACGCCCCGAACT	GCAGAAGAGCTGCTACGTGAA
p21 (*Cdkn1a*)	CCTGGTGATGTCCGACCTG	CCATGAGCGCATCGCAATC
Tnf	ATGAGAAGTTCCCAAATGGC	CTCCACTTGGTGGTTTGCTA
Il6	AACGATGATGCACTTGCAGA	TGGTACTCCAGAAGACCAGAGG
Il1b	TGAAGCAGCTATGGCAACTG	AGGTCAAAGGTTTGGAAGCA
Tgfb1	CAGCTCCTCATCGTGTTGGTG	GCACATACAAATGGCCTGTCTC
Il10	AAGGCAGTGGAGCAGGTGAA	CCAGCAGACTCAATACACAC
Arg1	CATGGGCAACCTGTGTCCTT	TCCTGGTACATCTGGGAACTTTC
Ccl2	GCCCCACTCACCTGCTGCTACT	CCTGCTGCTGGTGATCCTCTTGT
Ccl5	GCTGCTTTGCCTACCTCTCC	TCGAGTGACAAACACGACTGC
Cxcl1	CTGGGATTCACCTCAAGAACATC	CAGGGTCAAGGCAAGCCTC
Cxcl2	CCTGGTTCAGAAAATCATCCA	CTTCCGTTGAGGGACAGC
Cxcl5	GCTGCCCCTTCCTCAGTCAT	CACCGTAGGGCACTGTGGAC
S100a8	AAATCACCATGCCCTCTACAAG	CCCACTTTTATCACCATCGCAA
S100a9	TGACACCCTGAGCAAGAAGG	TGGTTTGTGTCCAGGTCCTC
Actb (β-Actin)	GGCTGTATTCCCCTCCATCG	CCAGTTGGTAACAATGCCATGT
