# Supplementary material for: Paquinimod Targeting of the S100A8/A9 Axis Suppresses Liver Metastasis in Aged Mice
Source: Cancers (Basel). 2026 May 19;18(10):1635. doi: 10.3390/cancers18101635 (PMC13204323; doi:10.3390/cancers18101635)
Supplement: Supplementary file 1 [file cancers-18-01635-s001.zip › Original Images for Blots v3.pdf]

Original Images for Blots

Figure 3.D

Figure 3.D

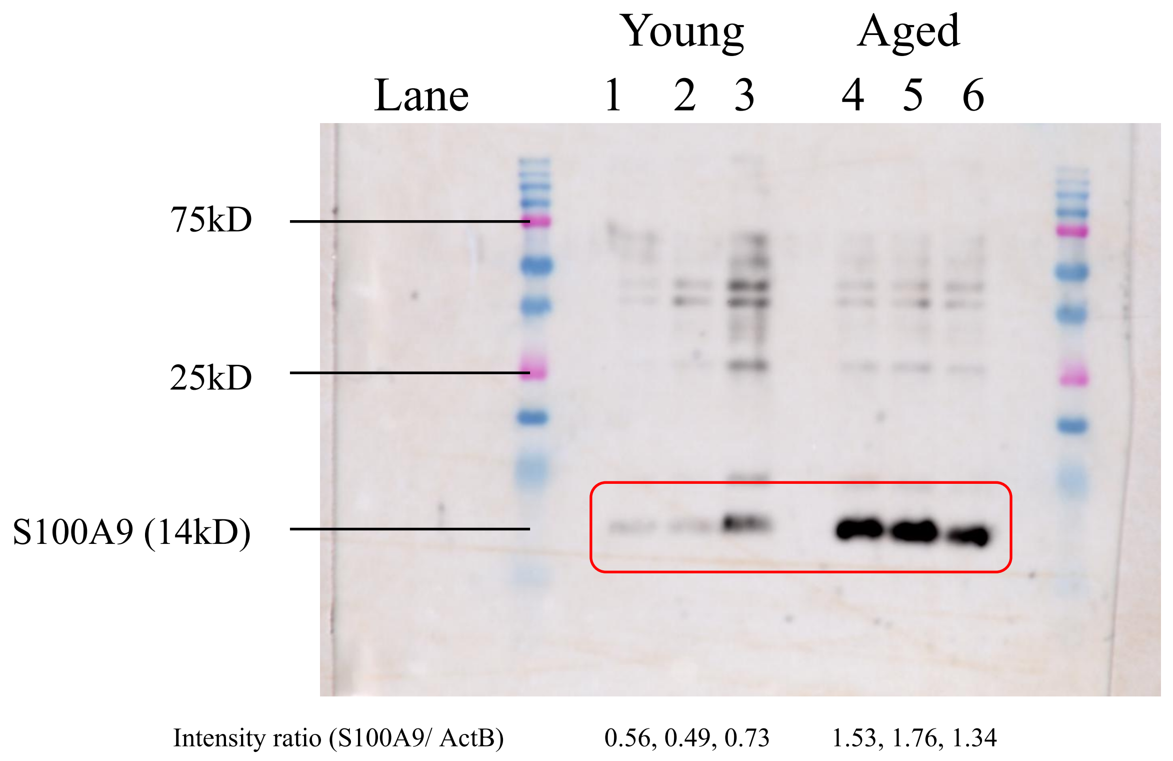

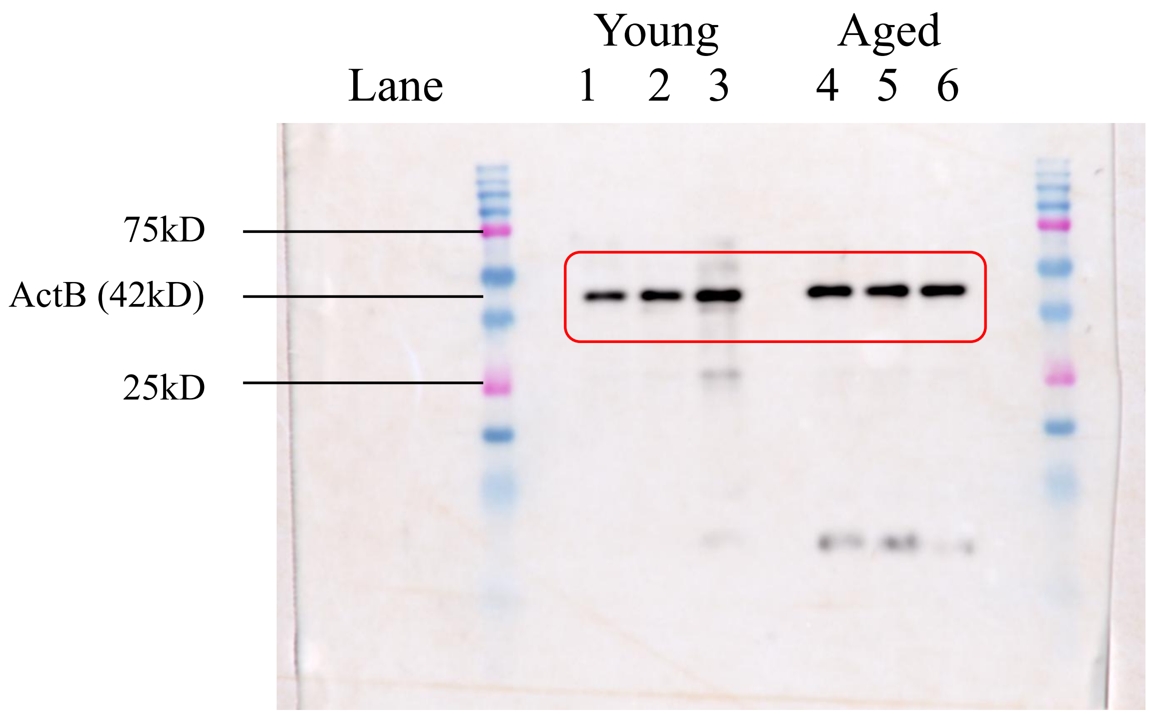

Lane1-3:Young, tumor-naïve livers. Lane4-6: Aged, tumor-naïve livers
The same membrane was probed for S100A9, stripped, and reprobed for β-actin (ActB).

Figure 3.E

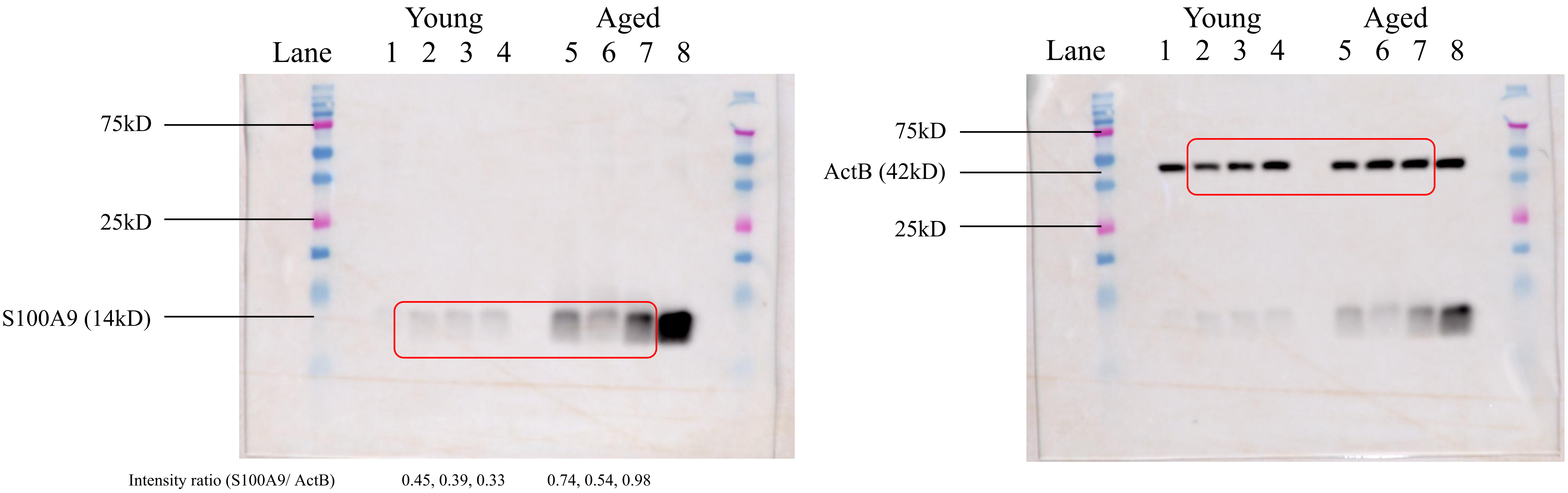

Lane1-4:Young, tumor. Lane5-8: Aged, tumor.
The same membrane was probed for S100A9, stripped, and reprobed for β-actin (ActB).

Figure S6

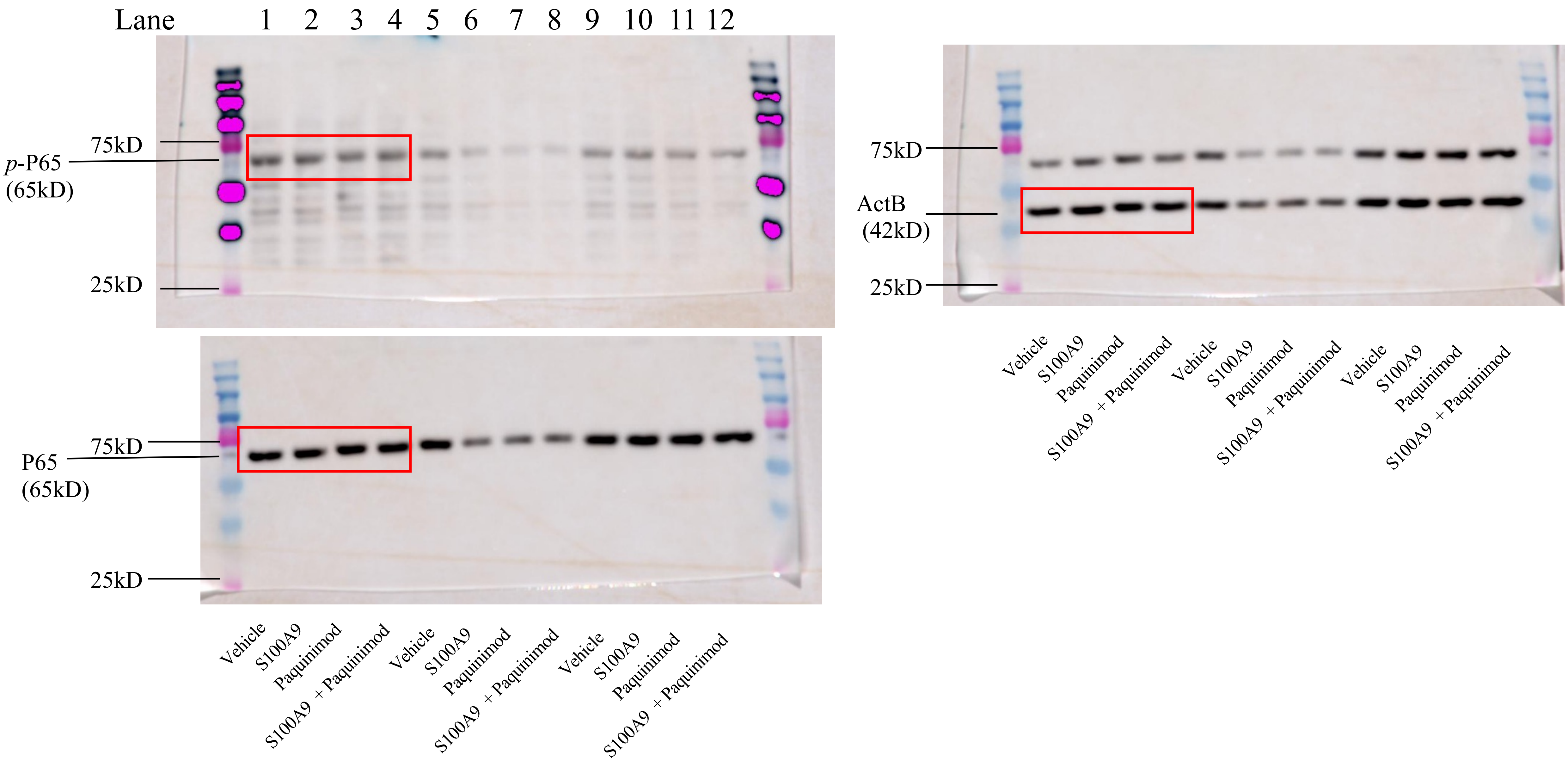

Lane 1 2 3 4 5 6 7 8 9 10 11 12

p-P65/ P65: 1.31, 1.20, 1.04, 1.10, 0.88, 1.05, 0.88, 0.86, 0.82, 0.85, 0.77, 0.75

Figure S7

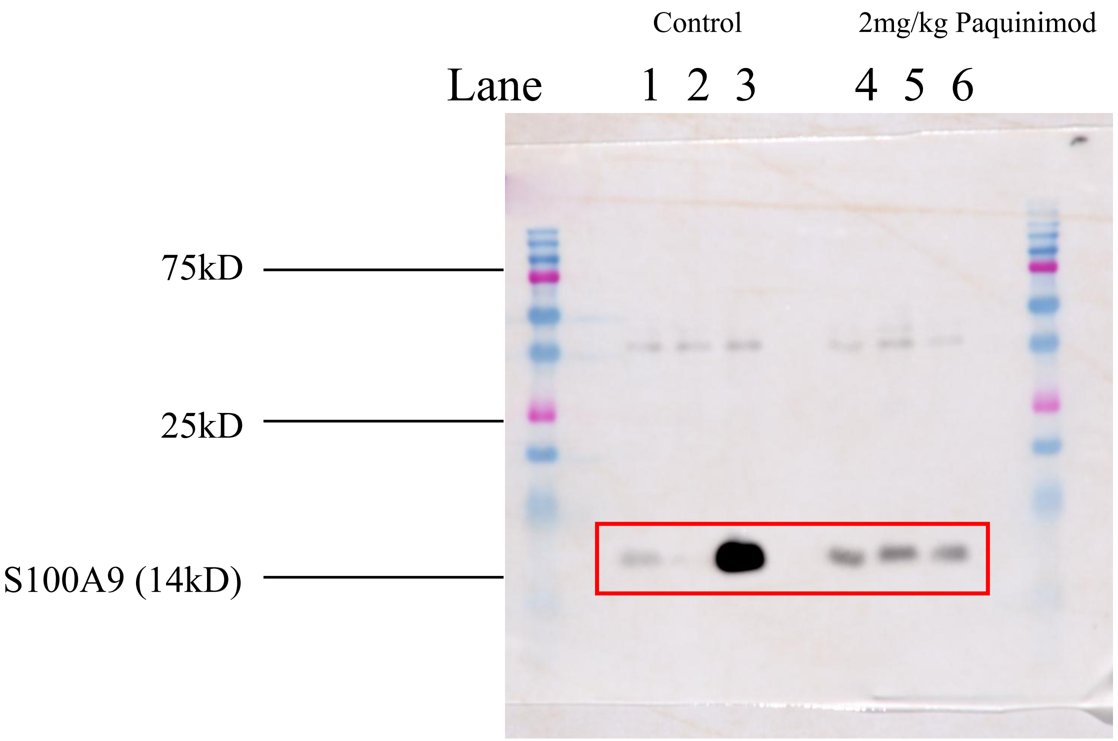

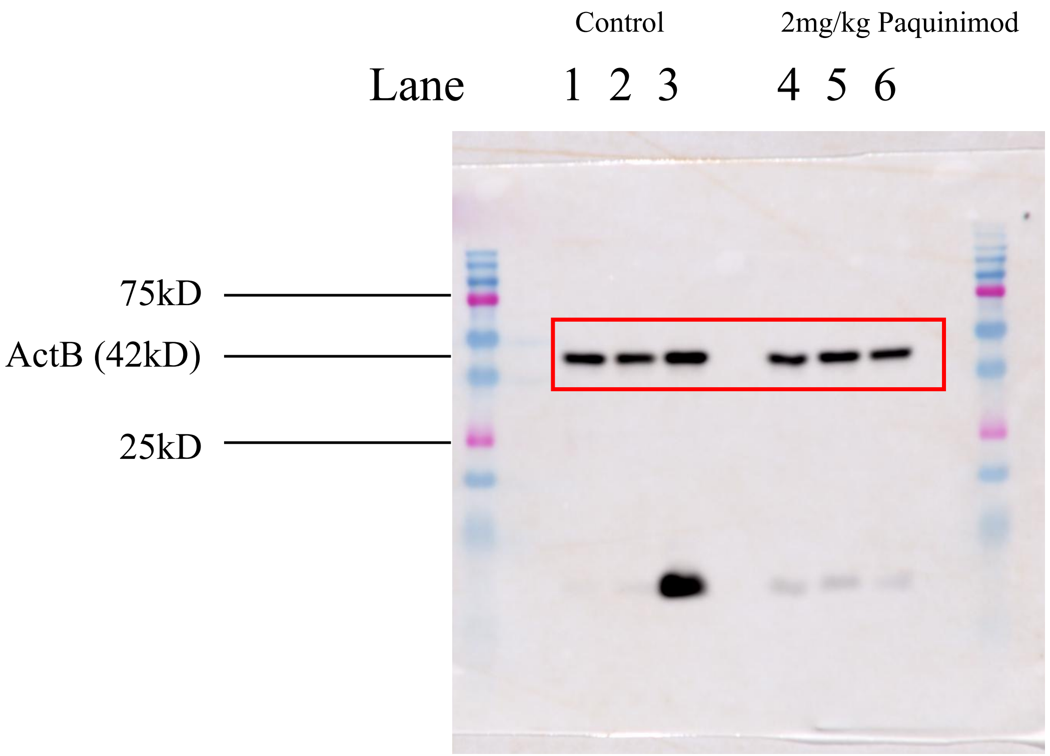

Intensity ratio (S100A9/ ActB) 0.47, 0.44, 2.08 0.73, 0.71, 0.66
